# After the Quacks in Van Zandt

**Published:** 1888-10

**Authors:** 


AFTER THE QUACKS.—We learn from a letter from a subscriber at Wills Point, VanZandt county, that "even under our defective laws, unsatisfactory as they are, violators of the medical act are being brought to time." A number have been convicted, and others have gone off to *low grade* colleges to graduate. One "Doctor Hartman," a notorious quack, was fined $200. The Medical Society of VanZandt county is "wide awake."

This is an example we should be pleased to see followed in every county. It shows one good, County Societies can do—prosecute the offenders, where an individual physician could not. Success to the VanZandt Society.

